# Prognostic Significance of lncRNA ATB and SNHG16 Expression Levels in Patients with Hepatocellular Carcinoma 

**DOI:** 10.30699/ijp.2025.2044199.3368

**Published:** 2025-10-30

**Authors:** Sahar Ravanshad, Mohammadhossein Taherynejad, Zohre Gerami, Amin Dalili, Hossein Ayatollahi, Hassan Mehrad-Majd

**Affiliations:** 1 *Department of Internal Medicine, Faculty of Medicine, Mashhad University of Medical Sciences, Mashhad, Iran*; 2 *Endoscopic and Minimally Invasive Surgery Research Center, Mashhad University of Medical Sciences, Mashhad, Iran*; 3 *Surgical Oncology Research Center, Mashhad University of Medical Sciences, Mashhad, Iran*; 4 *Cancer Molecular Pathology Research Center, Mashhad University of Medical Sciences, Mashhad, Iran *; 5 *Clinical Research Development Unit, Ghaem Hospital, Faculty of Medicine, Mashhad University of Medical Sciences*

**Keywords:** Hepatocellular Carcinoma, Long Noncoding RNAs, Tumor

## Abstract

**Background & Objective:**

Long noncoding RNAs (lncRNAs) are transcripts longer than 200 nucleotides, a major component of noncoding RNAs. Previous studies have shown the oncogenic role of various lncRNAs. This study aimed to explore the expression and prognostic value of ATB and SNHG16 in patients with hepatocellular carcinoma.

**Methods:**

ATB and SNHG16 expression in tumor and adjacent normal tissues was identified using real-time quantitative PCR. In this study, patients were divided into 2 groups based on the expression level of each gene, and the correlation between expression level and clinicopathological features of patients with HCC was investigated. All data were analyzed using SPSS version 22.

**Results:**

Of the lncRNAs examined in this study, ATB was expressed at significantly higher levels in tumor tissues. However, this overexpression was not associated with any clinicopathological features of HCC patients.

**Conclusion:**

In this study, overexpression of ATB and SNHG16 was not associated with survival rate or clinicopathological features of patients with hepatocellular carcinoma.

## Introduction

Hepatocellular carcinoma (HCC) is one of the most common cancers worldwide, accounting for more than 90% of primary liver tumors (1). Several risk factors are known for HCC, including HBV, HCV, obesity, diabetes mellitus, alcohol, chronic exposure to aflatoxin, and inherited disorders such as glycogen storage disease, chronic cholestatic syndromes, metal storage disease, and alpha-1-antitrypsin deficiency (2). In addition, 85% of patients with cirrhosis are diagnosed with HCC (3). With changing lifestyles and the increasing body mass index (BMI) as risk factors for cirrhosis, HCC is expected to become a global challenge (4). The mortality rate of HCC is also predicted to reach 1 million per year worldwide by 2030 (5, 6). Because the prognosis of patients with HCC depends on the stage at diagnosis and most patients are asymptomatic in the early stages, diagnosis at advanced stages poses another challenge (5, 7). Due to the rising risk of developing HCC, its risk factors, and its late diagnosis leading to poor prognosis, clinical outcome prediction, particularly in the early stages, is key to the treatment, management, and improved survival rate of patients. 

In recent decades, studies have focused on biomarkers as a valuable tool for earlier detection of various cancers. Alpha-fetoprotein (AFP) is a well-known and widely used biomarker in the diagnosis of HCC. However, studies have reported varying and contradictory results regarding the diagnostic utility of AFP, limiting its use (8-10). To date, several nucleic acid biomarkers have been introduced for HCC following genomic progression (11, 12). 

In humans, although 75% of the genome is transcribed into RNA, only 2% is translated into proteins. The remaining 98% of transcripts are noncoding RNAs (ncRNAs), which lack protein-coding ability (13). Long noncoding RNAs (lncRNAs) are a type of noncoding RNA consisting of transcripts longer than 200 nucleotides (14). Evidence indicates that lncRNAs have various functions in physiological and pathological processes, including cell proliferation, oncogenesis, and immunity. They are also essential in gene expression at various levels (15). Moreover, some lncRNAs have been found to be dysregulated in various diseases, such as cardiovascular disease, cancer, neurological diseases, and diabetes (16). 

Long noncoding RNA activated by transforming growth factor β (lncRNA-ATB) is located on chromosome 14 and is known as a cancer-related lncRNA (17). ATB promotes tumor development and alters cellular functions such as proliferation, migration, and invasion in various cancers by inducing epithelial-mesenchymal transition (EMT) and activating STAT3, ERK, and PI3K/AKT signaling pathways (17). 

Small nucleolar RNA host gene 16 (SNHG16), located on chromosome 17, is another oncogenic lncRNA involved in tumorigenesis. SNHG16, a member of the SNHG family, is upregulated in tumor tissues and correlated with metastasis and poor patient prognosis (18). Studies have shown that SNHG16 is overexpressed in osteoblastoma, lung cancer, HCC, breast cancer, cervical cancer, and bladder cancer (18). 

Despite efforts to study the genes and molecular biomarkers that are up- or downregulated in HCC patients, the biomarkers involved in HCC remain unclear. This study explores the expression of ATB and SNHG16 and their relationship with clinicopathological features in patients with HCC. 

## Materials and Methods

### Patients and Clinical Tissue Samples

Twenty-two pairs of cancerous tissue samples and adjacent normal tissue samples were collected from patients with a confirmed diagnosis of HCC by pathological tests. The patients were referred and admitted to Imam Reza and Montaserieh hospitals. Exclusion criteria included a history of chemotherapy or radiotherapy, and malignancy in other sites. After surgery, fresh tissue samples were cut into 0. 5-cm pieces and immediately placed in RNAlater (Thermo Fisher Scientific, Waltham, MA, USA) at 4°C overnight. For long-term storage, samples were moved and stored at -80°C until RNA extraction. 

This study was performed according to the principles of the Declaration of Helsinki and approved by the organizational ethics committee of the faculty of medicine at Mashhad University of Medical Sciences (MUMS) (code IR.MUMS.Medical.REC.1400.294). 

### Quantitative Real-Time Reverse Transcriptase PCR

According to the manufacturer's instructions, total RNA was extracted using an RNA extraction kit, Trizol Reagent (Sangon Biotech Co. Ltd. Shanghai, China). The extracted total RNA was evaluated at 260 nm using a NanoDrop device (Thermo-Fisher Scientific, Waltham, MA, USA). 

Subsequently, 2 μL of total RNA (20–200 ng) was reverse transcribed using a cDNA synthesis kit (Wizbiosolutions, Seongnam, Gyeonggi, Korea). 

The cDNA, gene primers (Table 1), SYBR Green master mix, and nuclease-free water were prepared in the Roche LightCycler® 96 System to perform quantitative real-time PCR (qRT-PCR) analysis. The experiments were repeated 3 times, and the 2^-ΔΔCt^ method was used to evaluate the expression level of the ATB and SNHG16 genes in relation to the reference gene GADPH. 

### Statistical Analysis

In this study, SPSS version 22 was used for data analysis. All variables were analyzed using central and dispersion indicators or frequency distribution, depending on their nature. The chi-square test and paired t-test were used to analyze data for qualitative and quantitative variables, respectively. For data that were not normally distributed, the Wilcoxon test was used. In addition, the Kaplan-Meier diagram was used to analyze overall survival. The Cox regression model was used in univariate and multivariate modes to determine the factors related to the survival rate. A P value <0.05 was considered significant for all analytical tests. 

### Ethical Considerations

All patients provided informed consent, and patient information was kept confidential. Patients were also given the option to withdraw from the study at any stage. 

## Results

### Demographic and Laboratory Data of Patients

The demographic and clinical characteristics of the 22 patients enrolled in this study are listed in Table 2. The average age of all patients was 52. 95 ± 15. 37 years, and 11 patients (50. 0%) were younger than 60 years. Nineteen patients (86. 4%) were male, and 14 (63. 6%) had liver cirrhosis. Ten patients (45. 5%) had HBV, and 2 (9. 1%) had HCV. Fifteen patients (68. 2%) had single nodules. Table 2 provides more detailed information. 

### ATB and SNHG16 Expression in HCC Tissues

**Fig. 1:**
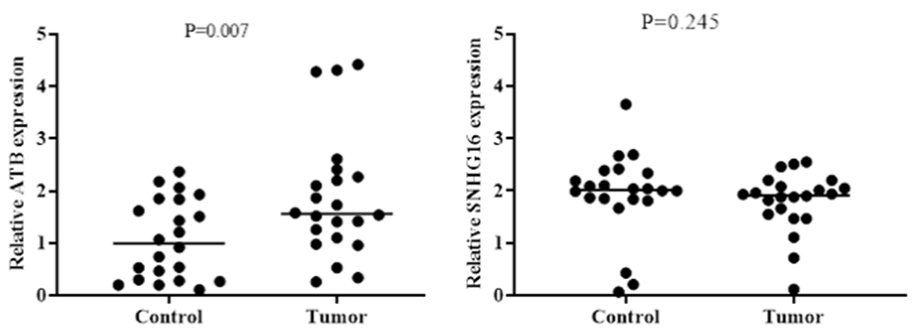
Expression of ATB and SNHG16 in HCC Tissue

After analysis, the expression level of ATB was significantly higher in tumor tissues than in normal tissues (P = .007). In contrast, the expression level of SNHG16 was not significantly different between tumor tissues and normal tissues. More information is shown in Figure 1. 

### Correlation Between ATB and SNHG16 Expression and Clinical Characteristics of Patients With HCC

Based on the expression level of each gene, patients were divided into 2 groups with high and low expression and compared in terms of different factors. Each group had 11 patients for the ATB gene. For the SNHG16 gene, 10 patients were classified into the high-expression group, and 12 patients were classified into the low-expression group. The relationship between ATB and SNHG16 gene expression and clinicopathological characteristics of patients with HCC is reported in Table 3. As shown, no statistically significant differences were found in the clinicopathological features between the 2 groups for either the ATB or SNHG16 genes. 

### Correlation of ATB and SNHG16 With Overall Survival of HCC Patients

**Fig. 2:**
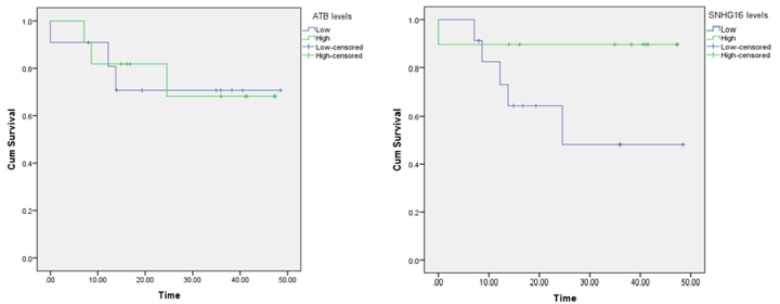
Results of Kaplan-Meier Analysis of ATB and SNHG16

The average overall survival time of patients in the high and low expression groups was 37. 10 ± 5. 03 years vs 36. 89 ± 5. 69 years for ATB, respectively, and 42. 66 ± 4. 49 years vs 31. 03 ± 5. 69 years for SNHG16. Furthermore, according to the log-rank test and the Kaplan-Meier survival curve shown in Figure 2, the differences in survival rates of ATB and SNHG16 genes based on the expression level were not statistically significant, with P values of 0.919 and 0.096, respectively.

**Table 1:** List of Primers Used in the Study

Gene	Forward primer	Reverse primer	Length
GAPDH	5'-TGCACCACCAACTGCTTA-3'	5'-GATGGCATGGACTGTGGTCAT-3'	90 bp
ATB	5'-TCTGGCTGAGGCTGGTTGAC-3'	5'-ATCTCTGGGTGCTGGTGAAGG-3'	142 bp
SNHG16	5'-GGACCCAAAGTGCCATGTCT-3'	5'-GATGAAGCCCAAAGAACGCA-3'	126 bp

**Table 2:** Demographic and Clinical Characteristics of Patients

Variables	Mean/ Frequency		% / SD
Age	**52** **.** **95**		**15** **.** **37**
Age distribution	**<60**	**11**	**50** **.** **0**
	**≥60**	**11**	**50** **.** **0**
Gender	**Female**	**3**	**13** **.** **6**
	**Male**	**19**	**86** **.** **4**
Tumor size	**5** **.** **18**		**2** **.** **60**
Liver cirrhosis	**Yes**	**14**	**63** **.** **6**
	**No**	**8**	**36** **.** **4**
HBV	**Yes**	**10**	**45** **.** **5**
	**No**	**12**	**54** **.** **5**
HCV	**Yes**	**2**	**9** **.** **1**
	**No**	**20**	**90** **.** **9**
Single nodule	**Single**	**15**	**68** **.** **2**
	**Multiple**	**7**	**31** **.** **8**
Tumor differentiation	**Well**	**10**	**45** **.** **5**
	**Moderate**	**8**	**36** **.** **4**
	**Poor**	**4**	**18** **.** **2**
Tumor encapsulation	**complete**	**15**	**68** **.** **2**
	**perforated**	**7**	**31** **.** **8**
Vascular invasion	**Yes**	**5**	**22** **.** **7**
	**No**	**17**	**77** **.** **3**
Tumor staging	**T1**	**8**	**36** **.** **4**
	**T2**	**3**	**13** **.** **6**
	**T3**	**4**	**18** **.** **2**
	**T4**	**7**	**31** **.** **8**
Liver transplant status	**Recipient**	**15**	**68** **.** **2**
	**Lobectomy**	**7**	**31** **.** **8**
Condition of patients	**Alive**	**16**	**72** **.** **7**
	**Expired**	**6**	**27** **.** **3**

**Table 3:** Relationship Between ATB and SNHG16 Expression and Clinicopathological Features in HCC Patients

**Variables**		**ATB Expression**		**SNHG16 Expression**	
		Low (n=11)	High (n=11)	Low (n=12)	High (n=10)
**Age (mean ±SD)**		**56.73 ** **±** ** 12.00**	**49.18 ** **±** ** 17.89**	**52.08 ** **±** ** 19.06**	**54.00 ** **±** ** 10.19**
**Tumor size**		**5.00 ** **±** ** 1.19**	**5.35 ** **±** ** 3.56**	**5.74 ** **±** ** 3.02**	**4.50 ** **±** ** 1.90**
**Age Composition**	<60	**5 (45.5)**	**6 (54.5)**	**5 (41.7)**	**6 (60.0)**
	≥60	**6 (54.5)**	**5 (45.5)**	**7 (58.3)**	**4 (40.0)**
**Gender**	Male (%)	**10 (90.9)**	**9 (81.8)**	**11 (91.7)**	**8 (80.0)**
	Female (%)	**1 (9.1)**	**2 (18.2)**	**1 (8.3)**	**2 (20.0)**
**Cirrhosis**	Yes	**6 (54.5)**	**8 (72.7)**	**7 (58.3)**	**7 (70.0)**
	No	**5 (45.5)**	**2 (27.3)**	**5 (30.0)**	**3 (30.0)**
**HBV**	Yes	**7 (63.6)**	**3 (27.3)**	**5 (41.7)**	**5 (50.0)**
	No	**4 (36.4)**	**8 (72.7)**	**7 (58.3)**	**5 (50.0)**
**HCV**	Yes	**0 (0.0)**	**2 (18.2)**	**1 (8.3)**	**1 (10.0)**
	No	**11 (100.0)**	**9 (81.8)**	**11 (91.7)**	**9 (90.0)**
**Tumor differentiation**	Well	**5 (45.5)**	**5 (45.5)**	**7 (58.3)**	**3 (30.0)**
	Moderate	**4 (36.4)**	**4 (36.4)**	**3 (25.0)**	**5 (50.0)**
	Poor	**2 (18.2)**	**2 (18.2)**	**2 (16.7)**	**2 (20.0)**
**TNM stage**	T1	**6 (54.5)**	**2 (18.2)**	**4 (33.3)**	**4 (40.0)**
	T2	**0 (0.0)**	**3 (27.3)**	**1 (8.3)**	**2 (20.0)**
	T3	**1 (9.1)**	**3 (27.3)**	**3 (25.0)**	**1 (10.0)**
	T4	**4 (36.4)**	**3 (27.3)**	**4 (33.3)**	**3 (30.0)**
**Tumor nodule**	Single	**9 (81.8)**	**6 (54.5)**	**8 (66.7)**	**7 (70.0)**
	Multiple	**2 (18.2)**	**5 (45.5)**	**4 (33.3)**	**3 (30.0)**
**Liver status**	Recipient	**8 (72.7)**	**7 (63.6)**	**8 (66.7)**	**7 (70.0)**
	Lobectomy	**3 (27.3)**	**4 (36.4)**	**4 (33.3)**	**3 (30.0)**
**Tumor encapsulation**	Complete	**7 (63.6)**	**8 (72.7)**	**8 (66.7)**	**7 (70.0)**
	Perforated	**4 (36.4)**	**3 (27.3)**	**4 (33.3)**	**3 (30.0)**
**Vascular invasion**	Yes	**3 (27.3)**	**2 (18.2)**	**2 (16.7)**	**3 (30.0)**
	No	**8 (72.7)**	**9 (81.8)**	**10 (83.3)**	**7 (70.0)**
**Patients condition**	Alive	**8 (72.7)**	**8 (72.7)**	**7 (58.3)**	**9 (90.0)**
	Expired	**3 (27.3)**	**3 (27.3)**	**5 (41.7)**	**1 (10.0)**

## Discussion

HCC is one of the most common human malignancies. In the past decade, despite various therapeutic strategies and preventive management of HCC patients, therapeutic performance results have been far from the expected therapeutic effect (19). Also, HCC incidence rates have increased along with its risk factors (20). Therefore, identifying new biomarkers to detect and predict the outcome of patients with HCC is a novel diagnostic and therapeutic approach to improving the survival rate of these patients. 

LncRNAs are known as transcription noise, but studies have shown that they influence a wide range of cellular processes. Moreover, they have a pathological role in disease processes, including various cancers, diabetes, and Alzheimer's disease (21-25). Although lncRNAs were once considered transcriptional noise, research has shown that they have a significant role in regulating numerous cellular processes. In addition, they contribute to the pathology of several diseases, including various cancers, diabetes, and Alzheimer disease. 

In this study, we explored the role of ATB and SNHG16 levels in clinicopathological characteristics and prognosis of HCC patients. Of the patients enrolled in this study, 16 (72. 7%) were still alive, and 6 (27. 3%) had died. Among the 2 lncRNAs investigated, only the expression of ATB in cancer tissues was significant. However, there was no significant relationship between the survival rate and the expression of these lncRNAs based on their expression. In addition, we examined the clinicopathological features of patients and their relationship with the 2 genes, but none of the relationships were significant. 

Some recent studies indicated that ATB, activated by TGF-β, promotes tumor progression, micro- and macrovascular metastasis, vascular invasion, and colonization of distant malignant cells by stimulating IL-11 secretion (26, 27). Furthermore, previous studies have shown that ATB is highly expressed in various cancers, including breast cancer, colorectal cancer, pancreatic cancer, and renal cell carcinoma (26, 28-32). Wang et al (33) reported that real-time PCR analysis revealed a significant increase in ATB expression in 72 cases of HCC tissues. The study also found a positive correlation with tumor size, TNM stage, and survival of HCC patients. Similar to that study, Jang et al (34) illustrated the significant expression of ATB in 100 cases of HCC tissues by real-time PCR. 

Compared with the current study, although ATB expression was significant in HCC tissues, there was no significant relationship with clinicopathological features or patient survival rate. Although the Kaplan-Meier survival curve shows no statistically significant difference in overall survival between ATB's high and low expression groups (P = 0. 919) and SNHG16 (P = 0. 096), a larger sample size could affect its statistical significance. The shape of the Kaplan-Meier curve of SNHG16 may suggest a trend that would reach statistical significance with a larger number of patients. In contrast, Zhang et al (35) claimed that SNHG16 is upregulated in HCC tissues and is an independent prognostic factor for HCC patients. Our study did not find significant survival differences for SNHG16 expression. However, this trend suggests that studies with larger cohorts may be worthwhile to validate its prognostic value. 

These different findings in previous studies compared with our study could be due to several reasons. First, the sensitivity and specificity of biomarkers vary in diverse populations, such as those of different age, sex, class, race, and ethnicity. Second, our study had some limitations, including a small sample size and lack of a control group, which may influence the accuracy of the results. Moreover, selection bias may occur because assessing the microvascular invasion or histological grade of the entire sample before liver biopsy is impossible. In addition, the expression of ATB and SNHG16 was evaluated only at the mRNA level. Assessing protein levels using IHC and western blot could have enhanced this study. 

## Conclusion

To date, the identification of potential lncRNA-mediated targets and mechanisms in various cancers has not been completed. We demonstrated that ATB expression was significantly increased in HCC. In addition to furthering the understanding of the pathogenesis of HCC, our findings will aid in identifying new lncRNA-based therapeutic strategies for these patients. 

## Data Availability

Data are available upon reasonable request from the corresponding author.
